# Characterization of Selective Antibacterial Peptides by Polarity Index

**DOI:** 10.1155/2012/585027

**Published:** 2012-04-01

**Authors:** C. Polanco, J. L. Samaniego, T. Buhse, F. G. Mosqueira, A. Negron-Mendoza, S. Ramos-Bernal, J. A. Castanon-Gonzalez

**Affiliations:** ^1^Centro de Investigaciones Químicas, Universidad Autónoma del Estado de Morelos, Avenida Universidad 1001, 62209 Cuernavaca, MOR, Mexico; ^2^Subdireccion de Epidemiologia Hospitalaria y Control de Calidad de la Atencion Medica, Instituto Nacional de Ciencias Medicas y Nutricion Salvador Zubiran, Vasco de Quiroga 15, Piso 4, Colonia Seccion XVI, 14000 Mexico City, DF, Mexico; ^3^Departamento de Matematicas, Facultad de Ciencias, Universidad Nacional Autonoma de México, Ciudad Universitaria, 04510 Mexico City, DF, Mexico; ^4^Direccion General de Divulgacion de la Ciencia, Universidad Nacional Autonoma de Mexico, Ciudad Universitaria, P.O. Box 70487, 04510 Mexico City, DF, Mexico; ^5^Instituto de Ciencias Nucleares, Universidad Nacional Autonoma de México, Ciudad Universitaria, P.O. Box 70543, 04510 Mexico City, DF, Mexico

## Abstract

In the recent decades, antibacterial peptides have occupied a strategic position for pharmaceutical drug applications and became subject of intense research activities since they are used to strengthen the immune system of all living organisms by protecting them from pathogenic bacteria. This work proposes a simple and easy statistical/computational method through a peptide polarity index measure by which an antibacterial peptide subgroup can be efficiently identified, that is, characterized by a high toxicity to bacterial membranes but presents a low toxicity to mammal cells. These peptides also have the feature not to adopt to an alpha-helicoidal structure in aqueous solution. The double-blind test carried out to the whole Antimicrobial Peptide Database (November 2011) showed an accuracy of 90% applying the polarity index method for the identification of such antibacterial peptide groups.

## 1. Introduction

The increasing resistance of pathogen agents towards multiple drugs has oriented parts of the investigation in bioinformatics to fast and efficient techniques that can predict the remarkable impact of antibacterial peptide action. These techniques can help to enhance the sometimes cumbersome chemical synthetic approach as well as the subsequent trial and error experiments to identify the peptide performance.

Among the proposed various classifications of peptides, one of it refers to the alpha-helicoidal versus beta-sheet conformation that the peptides can adopt in aqueous solution. This classification refers to the predominance of certain amino acids in the linear sequence of the peptides such as proline-arginine, cathelicidin, or cysteine. It is important to note that such classification appears to be without any influence on the toxicity or selectivity of the peptide once it got in contact with the target membrane [1, 2].

Although nature was used as the main source of peptides with antibacterial properties in the past [3], parts of the research efforts are now more directed towards synthetic strategies. One of these synthetic approaches generate the peptides by replacing and/or removing constitutive amino acids from a natural peptide known for its antibacterial action [4], thus trying to reduce its size while keeping or increasing its toxicity [5]. Another technique consists of joining two peptides that individually do not exhibit antibacterial properties but combined turn out to be highly toxic [6].

To obtain efficient antibacterial peptides by measuring the potential action of each altered peptide with the-above described methods would result in a possibility combination that exceeds by far the capacity of the known verification methods in the laboratory. For instance, the number of possible peptides to be formed from one peptide with 8 amino acids in length would be 20^8^ = 25,600,000,000 peptides. This is the reason why contemporary technique profiles to construct antibacterial peptides are the result of joint computational and/or mathematical methods to simulate peptide variations and then to evaluate and qualify these variations to eventually determine if the peptide complies with the required purposes. However, these methods with the aim to simulate the properties of the peptides as well as to evaluate their performance respecting all possible combinatorics are highly complex in their mathematical/computational model design.

In this paper, we present a statistical method that can be attributed to a single physical-chemical property, which is easy to computerize and that efficiently identifies antibacterial peptide subgroups for its highly selective toxicity to bacteria, hereinafter referred to as “Selective Cationic Amphipathic Antibacterial Peptides” (SCAAPs). A SCAAP is characterized by being less than 60 amino acids in length, not adopting an alpha-helicoidal structure in neutral aqueous solution, and showing a therapeutic index higher than 75 [7]. The therapeutic index of a peptide is defined as the ratio between the minimum inhibitory concentration observed against mammalian and bacterial cells [7, 8]; that is, the higher the value, the more specific the peptide for bacterial-like membranes. Hence SCAAPs display strong lytic activity against bacteria but exhibit no toxicity against normal eukaryotic cells such as erythrocytes [9].

Our method determines an index that we call polarity index that uses the existent 20 proteic amino acid classification differentiated by its side chain R that divides them in four types and three categories [10]. The three general categories of side chains are nonpolar, polar but uncharged, and charged polar. The nonpolar residues include those with aliphatic hydrocarbon side chains: Gly, Ala, Val, Leu, Ilu, Pro, one aromatic group, Phe, and one “pseudo-hydrocarbon,” Met. The polar but neutral category contains two hydroxyl-containing residues, Ser and Thr; two amides, Asn and Gln; two with aromatic rings, Tyr and Trp; one with a sulfhydryl group, Cys. In the charged polar class there are two amino acids with acidic groups, Asp and Glu, and three bases, His, Lys, and Arg (Table 1). The polarity index only makes use of that classification to get the SCAAP characteristic blueprint that in a double-blind test applied to all known peptides registered in the APD database (November 2011) [11] showed a very high efficiency.

## 2. Methods

### 2.1. Physicochemical Properties

Peptides can be expressed linearly as an amino acid sequence [12]. Such representation gives the peptide a unique blueprint. From this sequence, mathematical/computational algorithms have been designed with different complexity levels that measure a variety of physicochemical properties [13]. Among the properties on which the linear peptide representation focuses are two that define if a peptide falls into the category of SCAAP [7]; that is, when its measure meets simultaneously the parameters established for the following physicochemical properties:

- isoelectric point [14] (IP) from 9.65 to 11.80,
- hydrophobic moment [14] (HM) from 0.16 to 0.57.

Note that the original parameter values [7] have been extended. For this work, it was decided to take these two properties at a maximum range without considering the so-called AGADIR property, which is the tendency for not adopting an alpha-helicoidal structure in aqueous solution. As we have already verified [13], this property is not of significance for peptides with a length smaller than 22 amino acids.

A statistical-computational method was designed based only on one physicochemical property: polarity, which quickly and efficiently discerns if a peptide falls into the category of SCAAP or not. The verification was carried out by evaluating the IP and HM physicochemical properties.

### 2.2. Polarity Index Method

The polarity index method uses the 20 amino acid classification differentiated by their side chains that fall into four polarity groups: [P+] polar, [N] neutral, [P+] basic hydrophilic, and [NP] nonpolar residues (Table 1).

From these four groups, a polarity **P**[*i*, *j*] matrix is built with 16 elements that have as rows and columns the four different polarity groups set in the order P+, P−, N, NP and where **P**[*i*, *j*] matrix elements [*i*, *j*] represent the 16 possible interactions of the groups.

The method consists of the following steps.

- Creating a **P**[*i*, *j*] incidence matrix from the subject peptide.
- Generating a **Q**[*i*, *j*] incidence matrix from the SCAAP set.
- Comparing the incidences from both **P**[*i*, *j*] and **Q**[*i*, *j*] matrices.

### 2.3. **P**[*i*, *j*] Incidence Matrix from a Subject Peptide

The **P**[*i*, *j*] incidence matrix is built by adding to each of its elements the matches that occurred in the peptide subject sequence from the left to the right with two amino acids in length and by moving one amino acid to the right at the time until it arrives at the peptide side end. Each amino acid pair is related to its polarity group. From that association, we identify row *i* and column *j*. To the **P**[*i*, *j*] matrix element will be added 1, resulting thus in **P**[*i*, *j*] = **P**[*i*, *j*] + 1. Finally, the **P**[*i*, *j*] incidence matrix relative frequency distribution is normalized and weighted over a 0.30 factor. This last step helps to enhance the peptide distinctive characteristics by increasing the effect of the relative frequency position of the amino acids pairs in the incidence matrix **P**[*i*, *j*].

### 2.4. **Q**[*i*, *j*] Incidence Matrix from a SCAAP Set

The **Q**[*i*, *j*] incidence matrix is determined following the same procedure as for the **P**[*i*, *j*] incidence matrix. The peptide used here is the set of peptide sequences described in Table 2. The peptides used here as SCAAP templates were reported as SCAAP subjects by Del Rio et al. [7]. From the 7 peptides submitted, only those with a therapeutic index higher or equal to 1000 were chosen (Table 2, entries 3 and 7).

### 2.5. **P**[*i*, *j*] and **Q**[*i*, *j*] Matrices Comparison

In both the **P**[*i*, *j*] and the **Q**[*i*, *j*] matrices five stated positions M_1_, M_2_, M_3_, M_4_, and M_16_ were identified, where the subscript numeral stands for the element position in the matrix. The first row in the matrix represents the first four positions, the second row the next four positions, and so forth until allocating the last four positions to the last row. The position of the four elements with higher incidence would be M_1_, M_2_, M_3_, and M_4_ while M_16_ being the one with the lowest incidence. If the sequences {M_1_, M_2_, M_3_, M_4_, M_16._} for both matrices coincide, the peptide is classified as SCAAP.

### 2.6. Trial Data Preparation

1894 peptides registered in the Antimicrobial Peptide Database (APD) [11] (November 2011) were analyzed and classified by their single and multiple action against fungi, virus, mammalian cells, Gram+/Gram− bacteria, cancer cells, insects, parasites, and sperms. Peptides with more than one action were not included. The single action database only includes peptides with confirmed experimental action on a single pathogen agent, in contrast to multiple-action databases that contain peptides with action on two or more pathogen agents. On this basis, the figures in multiple action databases are over-represented.

The verification of peptides found in the single-action database on Gram+/Gram– bacteria was carried out by validating both the isoelectric point (IP) and hydrophobic moment (HM) in the ranges stated (see Section 2.1). The integrity of the APD database information was verified by checking identified peptides by their action in the whole extent of the database itself.

## 3. Results

Due to the importance of detecting possible peptide pathogenic action, the use of computer programs that evaluate peptic sequences to predict their action on different pathogen agents such as fungi, virus, mammalian cells, and Gram+/Gram– bacteria has become a standard practice among different research groups. The polarity index method is one of these computer programs, but it differs in measuring exclusively one physicochemical property to identify a SCAAP.

The **P**[*i*, *j*] Incidence matrix delivered by the polarity index method to identify a SCAAP used two peptides known by their toxic activity on Gram+/Gram+ bacteria (Table 2, entries 3 and 7) that turned out to be {M_1_, M_2_, M_3_, M_4_, M_16_} = {16, 4, 13, 15, 10}. SCAAP subjects identified from the provided single pathogenic action peptide database were fungi (0/77), viruses (0/22), mammalian cells (0/10), Gram+/Gram+ bacteria (51/743), cancer cells (1/16), insects (0/2), parasites (0/9), and sperms (0/0) (Table 3).

Note that the polarity index method only identified SCAAP subjects basically in the bacterial group. Whereas SCAAP subjects identified from the multiple pathogenic action peptide database were fungi (62/638), viruses (7/122), mammalian cells (20/205), Gram+/Gram+ bacteria (76/1489), cancer cells (21/121), insects (3/20), parasites (5/40), and sperms (1/9) (Table 3). Among the 743 peptides with a single action on Gram+/Gram– bacteria, the polarity index method identified 51 SCAAP subjects (Table 4), their IP and HM parameters were calculated and 46 of them are in the ranges previously mentioned in Section 2.1; that is, IP = 9.65–11.80 and HM = 0.16–0.57.

The APD database information integrity verification [11] showed 14 peptides not classified yet. When their activity as SCAAP was double checked by the polarity index method, there was a mismatch. The APD database margin of error did not exceed 8%.

## 4. Discussion

All different peptide classifications achieved over the decades seem to be directed to validate the peptide action and toxicity. However, it appears that these two characteristics are intrinsically related to the space where the peptide interacts as well as to the structural form of the subject membrane. Missing peptide specificity in the studied isolated peptides indicates that nature avoids peptide specificity in order not to favor certain pathogen agents in their blocking action.

Most peptides found experimentally show multiple actions on pathogen agents. Thus it appears that the detection and prediction of antibacterial peptides—in our case SCAAP—is more related to general, nonspecific peptide profiles that are well known for their antibacterial action. For that reason and as given in the present case, more efficient algorithms should rather evaluate fundamental characteristics of such peptides and search for small differences among them.

The design of bioinformatical algorithms to detect antimicrobial peptides is basically of two types.

- Based on a system of differential equations [15] that characterizes the peptide properties with an exponentially growing complexity.
- The inclusion of multiple peptide characteristics without affecting its complexity [16] where the efficiency greatly depends on a skillful peptide set selection.

Our polarity index method falls in the latter category and is characterized by the following.

- Effectively excluding multiple action peptides, with a margin of error less than 10% and single-action peptides with a margin of error less than 6%.
- Its efficiency to identify SCAAP subjects which is higher than 90%.
- The simplicity of the computational method which is easy to implement for massive parallel processing in GPUs [17].
- Its straightforwardness by measuring the peptide polarity exclusively and from this information effectively classifying its pathogenic action.

The algorithm involved in this method allows simple modifications to identify in a general level peptide groups by their pathogenic action and in a more specific level to refine the peptide search and identification as in the group used here.

The polarity index method uses the amino acid polarity classification; however there are other types of classifications [18, 19] that use the amino acid side chain chemical properties such as the neutral pH charge, their type of chemical structure, the reactivity, the elements present, or the ability to form hydrogen bonds. These classifications can be used to generate a more specific peptide blueprint when searched, with features that would not be considered otherwise.

As this method is a simple mathematical and computational algorithm, it does not demand heavy computational resources as processing memory or speed; therefore it can be used to explore peptide regions. These peptide regions can be worked out by evaluating massively all possible peptide combinations with the same length [20], thus taking advantage of the polarity index method simplicity to determine their activity.

## 5. Conclusion

The statistical/computacional polarity index method is an effective algorithm to find potential antibacterial peptides from a public domain database. These peptides have been denominated “Selective Cationic Amphipathic Antibacterial Peptides” (SCAAP). The method features a high efficiency to exclude peptides that exhibit single pathogenic action on other pathogens than bacteria, and it is equally efficient to exclude multiple-action peptides. In summary, the polarity index method is an adaptable and efficient method to detect and predict SCAAPs and it is a useful analysis and modeling tool for biological sequences using a single physicochemical property.

## 6. Availability

The polarity index computational implementation is listed in the Appendix section.

## Figures and Tables

**Table 1:** 20 proteinogenic amino acid classification differentiated by their side chain according to their polarity [10].

Symbol	Category	1-letter code
P−	polar	D, E, Y
N	neutral	C, G, N, Q, S, T
P+	basic hydrophilic	H, K, R
NP	non polar residues	A, F, I, L, M, P, V, W

**Table 2:** SCAAP subjects [7]. IP: estimated isoelectric point. HM: hydrophobic moment. TI: calculated therapeutic index. Peptides Cecropin-A and CA(1-8)M(1-18)NH2 were used to determine the P[*i*, *j*] incidence matrix.

Entry	Peptide	Sequence	IP	HM	TI
1	(KIAKKIA)2NH2	KIAKKIAKIAKKIA	11.5	0.48	86.2
2	(KLGKKLG)3NH2	KLGKKLGKLGKKLGKLGKKLG	11.7	0.49	98.3
**3**	**Cecropin-A**	**KWKLFKKIEKVGQNIRDGIIKAGPAVAVVGQATQIAK**	**11.2**	**0.44**	**1000.0**
4	Melittin	GIGAVLKVLTTGLPALISWIKRKRQQ	12.6	0.46	500.0
5	Magainin 2	GIGKFLHSAKKFGKAFVGEIMNS	10.8	0.56	75.0
6	CA(1-13)M(1-13)NH2	KWKLFKKIEKVGQGIGAVLKVLTTGL	11.1	0.53	400.0
**7**	**CA(1-8)M(1-18)NH2**	**KWKLFKKIGIGAVLKVLTTGLPALIS**	**10.4**	**0.43**	**2000.0**

**Table 3:** Number of matches in a typical SCAAP sequence in each peptide database with single or multiple action on fungi, viruses, mammalian cells, Gram+/Gram− bacteria, cancer cells, insects, parasites, and sperms (see also Section 2.6) [7].

Total	Action	Fungi	Viruses	Mammalian cells	Bacteria	Cancer cells	Insects	Parasites	Sperms
879	Unique	0/77	0/22	0/10	51/743	1/16	0/2	0/9	0/0
2644	Multiple	62/638	7/122	20/205	76/1489	21/121	3/20	5/40	1/9

**Table 4:** SCAAP subjects identified by the polarity index method in APD Gram+/Gram− bacteria database [11] where peptides have action only on bacteria. IP: estimated isoelectric point. HM: hydrophobic moment. Status: (X) not accepted for its IP and HM parameters, because the corresponding calculations were out of the ranges [7] (see Section 2.1).

No.	Peptide	Sequence	IP	HM	Status	Reference
1	Clavanin D (sea squirt, tunicate, invertebrates, animals)	AFKLLGRIIHHVGNFVYGFSHVF	10.85	0.54		[21]
2	Palustrin-1b (frog, amphibians, animals; XXU)	ALFSILRGLKKLGNMGQAFVNCKIYKKC	10.80	0.49		[22]
3	Palustrin-1d (frog, amphibians, animals; XXU)	ALSILKGLEKLAKMGIALTNCKATKKC	10.50	0.35		[22]
4	Palustrin-1c (frog, amphibians, animals; XXU)	ALSILRGLEKLAKMGIALTNCKATKKC	10.60	0.35		[22]
5	Brevinin-1PRc (frog, amphibians, animals; XXU)	FFPMLAGVAARVVPKVICLITKKC	10.50	0.38		[23]
6	Brevinin-1Be (frog, amphibians, animals; XXU)	FLPAIVGAAAKFLPKIFCVISKKC	10.30	0.43		[24]
7	Brevinin-1HSa (frog, amphibians, animals; XXU)	FLPAVLRVAAKIVPTVFCAISKKC	10.50	0.40		[25]
8	Brevinin-1Ba (frog, amphibians, animals; XXU)	FLPFIAGMAAKFLPKIFCAISKKC	10.30	0.50		[24]
9	Brevinin-1Bc (frog, amphibians, animals; XXU)	FLPFIAGVAAKFLPKIFCAISKKC	10.30	0.49		[24]
10	RANATUERIN 4 (ranatuerin-4, frog, amphibians, animals; XXU)	FLPFIARLAAKVFPSIICSVTKKC	10.50	0.46		[26]
11	Phylloseptin-H11 (PLS-H11, Phylloseptin-13, PS-13; frog, amphibians, animals; XXA)	FLSL IPHAINAVGVHAKHF	9.65	0.36		[27]
12	Phylloseptin-H5 (phylloseptin-7, PLS-H5, PS-7, XXA, frog, amphibians, animals)	FLSLIPHAINAVSAIAKHF	9.65	0.45		[28]
13	Phylloseptin-H2 (PLS-H2, Phylloseptin-2, PS-2) (XXA, frog, amphibians, animals)	FLSLIPHAINAVSTLVHHF	7.80	0.46	X	[29]
14	Phylloseptin-B1 (PLS-B1, PBN1; frog, amphibians, animals; XXA)	FLSLIPHIVSGVAALAKHL	9.65	0.46		[30]
15	Papilosin (tunicate, ascidian, invertebrates, sea animals)	GFWKKVGSAAWGGVKAAAKGAAVGGLNALAKHIQ	11.40	0.32		[31]
16	SMAP-34 (sheep myeloid antimicrobial peptide-34; OaMAP34, ovine cathelicidin, sheep, ruminant, animals)	GLFGRLRDSLQRGGQKILEKAERIWCKIKDIFR	10.43	0.48		[32]
17	Caerin 1.17 (frog, amphibians, animals; XXA)	GLFSVLGSVAKHLLPHVAPIIAEKL	9.50	0.49		[33]
18	Caerin 1.18 (frog, amphibians, animals; XXA)	GLFSVLGSVAKHLLPHVVPVIAEKL	9.50	0.50		[33]
19	Fallaxidin 3.2 (XXA, frog, amphibians, animals)	GLLDFAKHVIGIASKL	9.50	0.49		[34]
20	Fallaxidin 3.1 (XXA, frog, amphibians, animals)	GLLDLAKHVIGIASKL	9.50	0.48		[34]
21	Dahlein 5.2 (frog, amphibians, animals)	GLLGSIGNAIGAFIANKLKPK	11.10	0.52		[35]
22	Caerin 1.2 (XXA, frog, amphibians, animals)	GLLGVLGSVAKHVLPHVVPVIAEHL	7.02	0.49	X	[36]
23	Caerin 1.4 (XXA, frog, amphibians, animals)	GLLSSLSSVAKHVLPHVVPVIAEHL	7.02	0.48	X	[36]
24	Palustrin-2SIb (frog, amphibians, animals; XXU)	GLWNSIKIAGKKLFVNVLDKIRCKVAGGCKTSPDVE	10.10	0.36		[37]
25	XPF (the xenopsin precursor fragment, African clawed frog, amphibians, animals)	GWASKIGQTLGKIAKVGLKELIQPK	11.00	0.40		[38]
26	Pleurocidin (fish, animals)	GWGSFFKKAAHVGKHVGKAALTHYL	11.00	0.34		[39]
27	Cecropin (insects, invertebrates, animals)	GWLKKIGKKIERVGQNTRDATVKGLEVAQQAANVAATVR	11.30	0.36		[40]
28	Pm_mastoparan PMM (insects, invertebrates, animals; XXA; derivatives)	INWKKIASIGKEVLKAL	10.80	0.37		[41]
29	Hinnavin II (Hin II, insects, invertebrates, animals; JJsn)	KWKIFKKIEHMGQNIRDGLIKAGPAVQVVGQAATIYKG	10.12	0.45		[42]
30	Ostrich AvBD2 (Ostrich avian beta defensin 2, ostricacin-1, OSP-1, birds, animals; BBL)	LFCRKGTCHFGGCPAHLVKVGSCFGFRACCKWPWDV	8.94	0.33	X	[43]
31	Clavanin D (sea squirt, tunicate, invertebrates, animals)	LFKLLGKIIHHVGNFVHGFSHVF	10.80	0.56		[44]
32	Enterocin Q (EntQ, class 2d bacteriocins; leaderless, that is, no signal peptide, bacteria)	MNFLKNGIAKWMTGAELQAYKKKYGCLPWEKISC	10.00	0.39		[45]
33	Temporin-1Lb (Temporin 1Lb, frog, amphibians, animals)	NFLGTLINLAKKIM	10.80	0.41		[24]
34	Bovine beta-defensin 6 (bBD-6,cow, ruminant, animals)	QGVRNHVTCRIYGGFCVPIRCPGRTRQIGTCFGRPVKCCRRW	11.30	0.37		[46]
35	mBD-4 (mBD4, mouse beta-defensin 4, or Defb4, animals; 3S=S)	QIINNPITCMTNGAICWGPCPTAFRQIGNCGHFKVRCCKIR	8.91	0.33	X	[47]
36	ChBac5 (Pro-rich; Arg-rich, goat cathelicidin, ruminant, animals)	RFRPPIRRPPIRPPFNPPFRPPVRPPFRPPFRPPFRPPIGPFP	13.45	0.50		[48]
37	Cyclic dodecapeptide (OaDode, ovine cathelicidin, sheep, ruminant, animals)	RICRIIFLRVCR	12.00	0.36		[49]
38	Bactenecin (cyclic dodecapeptide, bovine cathelicidin, cow, cattle, ruminant, animals; BBMm; JJsn; derivatives: Bac2A)	RLCRIVVIRVCR	12.00	0.33		[50]
39	RL-37 (RL37, cathelicidin, Old World monkey, primates, animals)	RLGNFFRKVKEKIGGGLKKVGQKIKDFLGNLVPRTAS	11.90	0.59		[51]
40	BACTENECIN 7 (bac-7, bac 7; bac7; Pro-rich; cow cathelicidin, ruminant, animals; BBL, SeqAR, BBPP)	RRIRPRPPRLPRPRPRPLPFPRPGPRPIPRPLPFPRPGPRPIPRPLPFPRPGPRPIPRPL	13.20	0.25		[52]
41	Bac4 (Pro-rich, Arg-rich; cow cathelicidin, ruminant, animals)	RRLHPQHQRFPRERPWPKPLSLPLPRPGPRPWPKPL	12.90	0.23		[53]
42	Hyphancin IIIE (insects, invertebrates, animals)	RWKFFKKIERVGQNVRDGLIKAGPAIQVLGAAKAL	11.80	0.41		[54]
43	Cecropin B (insects, invertebrates, animals)	RWKIFKKIEKMGRNIRDGIVKAGPAIEVLGSAKAI	11.40	0.42		[55]
44	Hyphancin IIID (insects, invertebrates, animals)	RWKIFKKIERVGQNVRDGIIKAGPAIQVLGTAKAL	11.80	0.41		[54]
45	Hyphancin IIIG (insects, invertebrates, animals)	RWKVFKKIEKVGRHIRDGVIKAGPAITVVGQATAL	11.80	0.38		[54]
46	Hyphancin IIIF (insects, invertebrates, animals)	RWKVFKKIEKVGRNIRDGVIKAGPAIAVVGQAKAL	11.80	0.39		[54]
47	Phylloseptin-H4 (Phylloseptin-6, PLS-H4, PS-6, XXA, frog, amphibians, animals)	SLIPHAINAVSAIAKHF	9.65	0.47		[29]
48	Pep5 (Lantibiotic, type 1, class 1 bacteriocin, Gram-positive bacteria; XXT3; XXW3)	TAGPAIRASVKQCQKTLKATRLFTVSCKGKNGCK	11.10	0.27		[56]
49	Clavanin C (sea squirt, tunicate, invertebrates, animals)	VFHLLGKIIHHVGNFVYGFSHVF	9.55	0.47		[21]
50	Andropin (insects, invertebrates, animals)	VFIDILDKVENAIHNAAQVGIGFAKPFEKLINPK	7.50	0.45	X	[57]
51	Clavanin A (urochordates, sea squirts, and sea pork, tunicate, invertebrates, animals)	VFQFLGKIIHHVGNFVHGFSHVF	9.71	0.61		[21]

## Appendix

### Source Program for the Detection of SCAAP by the Polarity Index Method

c  Author Carlos Polanco 2011.
c  Program Detection of SCAAP by Polarity-index method.
c  Operating System: GNU Linux Fedora 14
c  Compilation: gfortran program. f
c  Execution:  ./a.out AEVAPAPAAAAPAKAPKKKAAAKPKKAGPS
 implicit none
 character ∗ 1 arreglo(100), arreglo3(500)
 character ∗ 500 backup
 character ∗ 1 convert
 integer convertN, tipo2
 integer base(16), candidato(16), aciertos2, aciertos0
 integer aciertost, aciertos3, aciertos4, aciertos14, aciertos24
 integer aciertos34, aciertos44, aciertos04, aciertos1, aciertos5
 integer x1, x2, x3, x4, *n*, *j*, *i*, *k*
 real tipo1
 real comodin
 double precision matriz(4, 4)
 double precision total, peso(4, 4)
 equivalence (arreglo3, backup)
 open (2, file = “candidate0.dat”)
34  format (f8.4, 1x, I2)
52  format (A3)
c  Relative frequency position of pairs of amino acid in the
c  candidate SCAAP
 peso (4, 4) = 0.272727281/0.272727281
 peso (1, 4) = 0.209790215/0.272727281
 peso (4, 1) = 0.164335668/0.272727281
 peso (1, 1) = 0.087412588/0.272727281
 peso (4, 3) = 0.083916083/0.272727281
 peso (3, 3) = 0.062937066/0.272727281
 peso (3, 4) = 0.059440561/0.272727281
 peso (3, 1) = 0.024475524/0.272727281
 peso (2, 1) = 0.006993007/0.272727281
 peso (1, 3) = 0.006993007/0.272727281
 peso (4, 2) = 0.006993007/0.272727281
 peso (2,4) = 0.003496503/0.272727281
 peso (2, 3) = 0.003496503/0.272727281
 peso (1, 2) = 0.003496503/0.272727281
 peso (3, 2) = 0.003496503/0.272727281
 peso (2, 2) = 0.000000000/0.272727281
c  Position of pairs of amino acid in the candidate SCAAP
 base(1) = 16
 base(2) = 4
 base(3) = 13
 base(4) = 15
 base(5) = 12
 base(6) = 1
 base(7) = 11
 base(8) = 9
 base(9) = 3
 base(10)= 14
 base(11)= 6
 base(12)= 8
 base(13)= 2
 base(14)= 7
 base(15)= 5
 base(16)= 10
 do *i* = 1, 4
  do *j* = 1, 4
  matriz (*i*, *j*) = 0
  enddo
 enddo
 x1 = 0
 x2 = 0
 x3 = 0
 x4 = 0
 total = 0
*k* = 0
*n* = 0
c  Command to gets the peptide (sequence of amino acid in letter-code)
 call getarg (1, backup)
 do *i* =1,500
  if (arreglo3(i). ne. “ ”) *n* = *n* + 1
 enddo
 do *i* = 1, *n*
  arreglo (*i*) = convert(arreglo3(*i*))
 enddo
c  Procedure to determine the relative frequency
c  distribution of amino acid in the sequence
 do *i* = 1, (*n* − 1)
  if (arreglo(i).eq. “1”) x1 = x1 + 1
  if (arreglo(i).eq. “2”) x2 = x2 + 1
  if (arreglo(i).eq. “3”) x3 = x3 + 1
  if (arreglo(i).eq. “4”) x4 = x4 + 1
  if (arreglo(i).eq. “0”) goto 100
  if (arreglo(i).ne. “0”) total = total +1
  matriz (convertN (arreglo (*i*)), convertN (arreglo (*i* + 1))) =
& matriz (convertN (arreglo (*i*)), convertN (arreglo (*i* + 1))) + 1
 enddo
100  do *i* = 1, 4
    do *j* = 1, 4
*k* = *k* + 1
   write (2,34)  (matriz(*i*, *j*)∗peso(*i*, *j*)∗∗1.3)/total, *k*
  enddo
 enddo
 close(1)
 close(2)
 call system (“sort −r candidate0.dat > candidate1.dat”)
 open (3, file = “candidate1.dat”)
 open (4, file = “candidate0.dat”)
 do *i* = 1, 16
  read (3, ∗) tipo1, tipo2
  write (4, ∗) tipo2
 enddo
 close(3)
 close(4)
 open (2, file = “candidate0.dat”)
c  Procedure to evaluate if the sequence of peptide is or
c  not candidate SCAAP
 do *i* = 1, 16
 read (2, ∗, END = 101) candidato(*i*)
 enddo
 call parte04 (base, candidato, aciertos0)
 call parte14 (base, candidato, aciertos1)
 call parte54 (base, candidato, aciertos5)
 if ((aciertos0.eq.1). and.(aciertos1.eq.3).and. (aciertos5.eq.1))then
  write (6, 52) “Yes”
 else
  write (6, 52) “No”
 endif
 call system (“rm candidate0.dat”)
 call system (“rm candidate1.dat”)
101  stop
 end
c  Subroutines and functions
c  Verification of position 1
 subroutine parte04(base, candidato, aciertos0)
 integer base(16), candidato(16), aciertos0
 aciertos0 = 0
 if (candidato(1).eq. base(1)) aciertos0 = aciertos0 + 1
 return
 end
c  Verification of positions 2, 3 and 4
 subroutine parte14(base, candidato, aciertos1)
 integer base(16), candidato(16), aciertos1
 aciertos1 = 0
 do *i* = 2, 4
 if (candidato(*i*).eq. base(*i*)) aciertos1 = aciertos1 + 1
 enddo
 return
 end
c  Verification of position 16
 subroutine parte54 (base, candidato, aciertos5)
 integer base(16), candidato(16), aciertos5
 aciertos5 = 0
 if (candidato(16).eq. base(16)) aciertos5 = aciertos5 + 1
 return
 end
c  Conversion letters to the corresponding groups of polarity (in numbers)
 character function convert(tipo)
 character ∗ 1 tipo
 if (tipo.eq. “A”) convert = “4”
 if (tipo.eq. “C”) convert = “3”
 if (tipo.eq. “D”) convert = “2”
 if (tipo.eq. “E”) convert = “2”
 if (tipo.eq. “F”) convert = “4”
 if (tipo.eq. “G”) convert = “3”
 if (tipo.eq. “H”) convert = “1”
 if (tipo.eq. “I”) convert = “4”
 if (tipo.eq. “K”) convert = “1”
 if (tipo.eq. “L”) convert = “4”
 if (tipo.eq. “M”) convert = “4”
 if (tipo.eq. “N”) convert = “3”
 if (tipo.eq. “P”) convert = “4”
 if (tipo.eq. “Q”) convert = “3”
 if (tipo.eq. “R”) convert = “1”
 if (tipo.eq. “S”) convert = “3”
 if (tipo.eq. “T”) convert = “3”
 if (tipo.eq. “V”) convert = “4”
 if (tipo.eq. “W”) convert = “4”
 if (tipo.eq. “Y”) convert = “2”
 if (tipo.eq. “X”) convert = “0”
 return
 end
c  Conversion number in code-letters to numbers in code-numbers
 integer function convertN(tipo)
 character ∗ 1 tipo
 if (tipo.eq. “1”) convertN = 1
 if (tipo.eq. “2”) convertN = 2
 if (tipo.eq. “3”) convertN = 3
 if (tipo.eq. “4”) convertN = 4
 return
 end
