# Erratum to “Characterization of Selective Antibacterial Peptides by Polarity Index”

**DOI:** 10.1155/2012/613053

**Published:** 2012-12-24

**Authors:** C. Polanco, J. L. Samaniego, T. Buhse, F. G. Mosqueira, A. Negron-Mendoza, S. Ramos-Bernal, J. A. Castanon-Gonzalez

**Affiliations:** ^1^Centro de Investigaciones Químicas, Universidad Autónoma del Estado de Morelos, Avenida Universidad 1001, 62209 Cuernavaca, MOR, Mexico; ^2^Subdireccion de Epidemiologia Hospitalaria y Control de Calidad de la Atencion Medica, Instituto Nacional de Ciencias Medicas y Nutricion Salvador Zubiran, Vasco de Quiroga 15, Piso 4, Colonia Seccion XVI, 14000 Mexico City, DF, Mexico; ^3^Departamento de Matematicas, Facultad de Ciencias, Universidad Nacional Autonoma de México, Ciudad Universitaria, 04510 Mexico City, DF, Mexico; ^4^Direccion General de Divulgacion de la Ciencia, Universidad Nacional Autonoma de Mexico, Ciudad Universitaria, A.P. 70-487, 04510 Mexico City, DF, Mexico; ^5^Instituto de Ciencias Nucleares, Universidad Nacional Autonoma de México, Ciudad Universitaria, A.P. 70-543, 04510 Mexico City, DF, Mexico

When I was using the Polarity-Index method a few days ago, I noticed differences in Table 3, as published.

However, it should be as in Table 3.

These changes do not affect neither the efficiency of the method nor any section of the paper.

I also noted the difficulty to compare information on the same APD2 database (November 2011), as it changes weekly. Therefore I am sending a file to support that test, in case the journal has backup for additional information. Otherwise I will to keep this information for three years. 

## Supplementary Material

Test files represent the APD2 (November 2011).

## Figures and Tables

**Table 1:** Number of matches in a typical SCAAP sequence in each peptide database with single or multiple action on fungi, viruses, mammalian cells, Gram+/Gram− bacteria, cancer cells, insects, parasites, and sperms (see also Section 2.6) [7].

Total	Action	Fungi	Viruses	Mammalian cells	Bacteria	Cancer cells	Insects	Parasites	Sperms
879	Unique	0/77	0/22	0/10	62/743	1/16	0/2	0/9	0/0
2644	Multiple	72/638	6/122	33/205	152/1489	11/121	6/20	5/40	1/9

